# Supplementary material for: AB5-Type Toxin as a Pentameric Scaffold in Recombinant Vaccines against the Japanese Encephalitis Virus
Source: Toxins (Basel). 2023 Jun 29;15(7):425. doi: 10.3390/toxins15070425 (PMC10467048; doi:10.3390/toxins15070425)
Supplement: Supplementary file 1 [file toxins-15-00425-s001.zip › toxins-2459491-supplementary.pdf]

Supplementary Figure S1

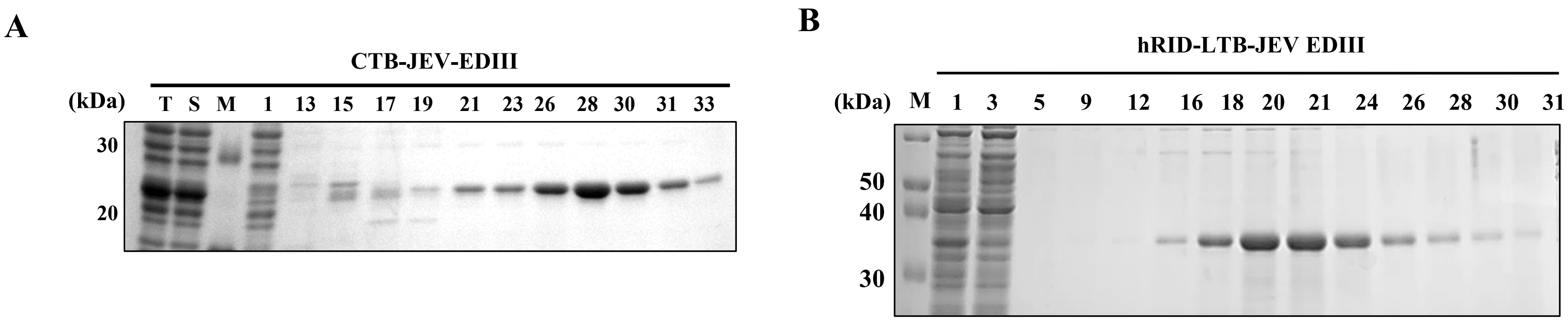

C

Protein	Total yield in 500 ml (mg)
CTB-JEV-EDIII	0.75
hRID-LTB-JEV-EDIII	2.70

Supplementary Figure S1. Purification of CTB-conjugated JEV EDIII by nickel chromatography. (A, B) CTB-JEV EDIII (A)and hRID-LTB-JEV EDIII (B) were separated by 8-15% SDS-PAGE gel after purification. Numbers on each lane represents eluted fraction number by gradient concentration of imidazole. (C) Estimated protein concentrations after his-tagged chromatography.
